# A description of a hepatitis A outbreak in men who have sex with men and public health measures implemented in Seine-Maritime department, Normandy, France, 2017

**DOI:** 10.1186/s12889-020-09499-3

**Published:** 2020-09-22

**Authors:** Nathalie Nicolay, Maggie Le Bourhis-Zaimi, Anais Lesourd, Mélanie Martel, Anne-Marie Roque-Afonso, Stéphane Erouart, Manuel Etienne, Ndeindo Ndeikoundam Ngangro

**Affiliations:** 1 Santé publique France, French national public health agency, Normandy regional office, 76100 Rouen, France; 2 European Centre for Disease Prevention and Control (ECDC), Gustav III:s boulevard 40, 16973 Solna, Sweden; 3 Infectious and Tropical Diseases Department, Rouen University Hospital, 76000 Rouen, France; 4 National reference laboratory for hepatitis A virus, 94804 Villejuif, France; 5 Regional Health directorate, 14000 Caen, France; 6 Normandie Univ, UNIROUEN, UNICAEN, GRAM 2.0, 76000 Rouen, France; 7 Santé publique France, French national public health agency, 94415 Saint-Maurice, France

**Keywords:** Outbreak, Hepatitis a, MSM, Sexually transmitted infection, Vaccination, Prevention

## Abstract

**Background:**

In 2016–2017, a European-wide circulation of genotype IA hepatitis A virus was responsible for hepatitis A outbreaks in men who have sex with men (MSM). This study aimed to describe the outbreak investigation in Seine-Maritime department (France) and the control measures implemented accordingly.

**Methods:**

Outbreak description used data from mandatory reporting and enhanced surveillance of male cases. Confirmed case was genotype IA isolated, possible cases had no reported genotype information. Targeted control measures included communication on sexual practices at risk of hepatitis A transmission and two vaccination campaigns in April 2017 and January 2018. Characteristics of cases and vaccinees were described. We reported the best communication channel for relaying outbreak information and control measures based on the monitoring of social network activities and feedback from vaccinees.

**Results:**

During the outbreak period (December 2016 to December 2017), a total of 48 confirmed outbreak cases and 30 possible outbreak cases were notified. Among them, 69 were male (88%). Two epidemic waves were observed. Cases encountered their partners through gay-dating apps (54%) and in one specific sauna (62%). In response to the outbreak, two vaccination campaigns were deployed. A total of 156 MSM were vaccinated, of whom 56 in a truck parked beside the sauna. Most of the vaccinees had been informed about the campaign through dating apps (44%). Community-based organizations involved in sexual health promotion and other gay social media were very proactive in sharing information about the outbreak and promoting the vaccination campaign through their social media account and also on site (gay venues). Vaccinees reported the same sexual practices at risk of hepatitis A transmission as cases.

**Conclusions:**

In response to this massive hepatitis A outbreak that affected mostly MSM in Seine-Maritime department, vaccination campaign remained the cornerstone of prevention. Prevention officers from the community-based organization played a key role in vaccination promotion. Gay-dating apps and outdoor sessions of vaccination allowed to effectively reach MSM. Cost-effectiveness studies might analyze the interest of a continuous sexual health promotion including vaccination against hepatitis A in MSM through dating apps and social networks.

## Background

Hepatitis A is an acute viral infection caused by the Hepatitis A Virus (HAV). The mean incubation period is about 28 days but ranges between 15 and 50 days [1]. While often asymptomatic in children, jaundice may occur in up to 70% of adult cases and fulminant hepatitis in 1% of them [1]. The reservoir of HAV is exclusively human and the transmission route is predominantly oro-fecal through the ingestion of contaminated water or food product and/or closed contact with an infected person. The period of infectivity usually starts 1 to 2 weeks before the onset of illness [1], while cases are still asymptomatic which is an aggravating factor for hepatitis A transmission and control.

In high income countries, hepatitis A is reported in travelers back from endemic areas, in foodborne outbreaks or in outbreaks that occur in closed settings (e.g school, crèches and minority ethnic groups) [2]. Hepatitis A is also commonly considered a sexually transmitted infection (STI) in men who have sex with men (MSM) with many reports since early 2000 [3–7]. During sexual intercourses, contamination may occur during oral–anal, digito–anal and genito–oral sex with an infected partner [8, 9]. Venues for casual sex, such as gay saunas and darkrooms have been implicated in large outbreaks [3, 10–12]. Outbreaks that occur MSM may spread to the general population (« spillover ») through contacts with infected persons [13].

Overall, improvement of hygiene largely contributed to the decreasing trend in hepatitis A seroprevalence in the general population in Europe. Seroprevalence studies showed that seroconversion increased with age but the level of seroconversion varies in Europe (higher in the south and eastern part) [14]. As a consequence susceptibility to infection may be high in young (male) adults despite that prevalence in MSM could exceed the one in the general population (42% versus 16% in an old report [15]). Early report described that the incidence in MSM was correlated with the number of sexual partners [16].

Prevention of HAV transmission during sexual intercourses relies on extremely careful personal hygiene e.g. washing hands and genital areas before and after sex. The use of dental dams for oral-anal sex and of latex gloves during digito-anal sex may offer protection. Vaccine is the most effective protection against hepatitis A with specific recommendations in MSM in France and elsewhere in Europe [1, 13]. The World Health Organization does not provide recommendations on the use of hepatitis A vaccination for outbreak control although immunization has been reported to be effective in controlling outbreaks in small communities [17]. The use of condoms is ineffective against the faecal-oral transmission route but offers protection against other STIs such as HIV during anal and oral-anal sex [13].

In 2017, an outbreak extensively affected MSM throughout Europe [18–24]. Early publications reported that three HAV strains of genotype IA (genotype sequences VRD_521_2016, RIVM-HAV16–090 or V16–25801) circulated in several European Union/ European Economic Area (EU/EEA) member States since June 2016 [25–28]. The European Center for Disease Prevention and Control (ECDC) first reported on the circulation of these strains in December 2016 and the total number of confirmed outbreak cases reported from June 1, 2016, up to September 7, 2018, reached 4475 [29].

We hereby aimed to describe the investigation and the public health control measures implemented in response to the notification of a cluster of hepatitis A cases in male in December 2016.

## Methods

### Seine maritime department

Seine-Maritime department (1,200,000 inhabitants) is one of the five departments of the Normandy region in northern France. It encompasses two main cities: Rouen and its metropolitan area (500,000 inhabitants), the capital of the Normandy region, and Le Havre (100,000 inhabitants) (www.insee.fr). General practitioners and STIs clinics (two in Rouen and one in Le Havre) are in frontline to provide clinical care and sexual health counselling. Previous hepatitis A outbreaks were reported in the Rom population [30].

### Outbreak detection

In week 02–2017, the first cluster associated with the European outbreak was notified to the health authorities in Seine-Maritime department. It consisted of 5 male cases with onset symptoms between week 50–2016 to week 01–2017 [31]. One of these men spontaneously reported homosexual intercourses with an HAV-infected partner (exposure not routinely collected in the notification form) during a sex-party.

### Description of the outbreak

#### Case definition

A case of hepatitis A was defined as any person aged 18 years or more, with a positive detection of anti-hepatitis A virus IgM antibodies from December 2016 to December 2017, in Seine-Maritime department. Cases were subsequently classified according to isolation or not of European outbreak strains as 1) confirmed outbreak case if genotype IA was isolated whether or not the genetic sequence was available (genotype sequences VRD_521_2016, RIVM-HAV16–090 or V16–25801), 2) possible outbreak case if no further information on genotyping was available, 3) unrelated case if another genotype than genotype IA was identified.

#### Enhanced surveillance

Since 2005, HAV surveillance relies on a mandatory notification by clinicians and laboratories [1]. At case notification, the public health nurse from the regional health directorate collects details on possible source of contamination and closed contacts using a standard questionnaire. Information on sexual exposure is not collected in routine. Cases receive counselling on good personal hygiene. Vaccination of contact is usually recommended.

In January 2017, upon request from the regional health directorate, the regional public health agency was tasked to better describe the characteristics of the outbreak in order to identify a profile of cases at risk and to target public health control measures. In this aim, from January 2017 onward, the epidemiologists from the regional public health agency proposed to enhance the epidemiological surveillance of hepatitis A cases with documentation of homosexual status in male cases. Subsequently, any identified MSM case was questioned about his sexual practices (steady and casual partners, anonymous sex, group sex), place of sexual encounters (e.g gay sauna attendance), means of sexual encounters (e.g use of dating apps), history of Human Immunodeficiency Virus (HIV) testing, and travel abroad with possible exposure to HAV including homosexual relationships in the past 2 months prior to diagnosis.

#### Virological surveillance

The National Hepatitis A Reference laboratory performed genotyping as previously described [32]. Analysis were performed each month on a subset of sera due to the flow of request received by the reference laboratory.

### Public health control measures and data collected

#### Vaccination campaign

In France, hepatitis A vaccination is recommended in MSM since 2002 [33]. It is offered for free of charge in STIs clinics. The regional health directorate has the mandate to implement control and prevention measures in case of health threat. When the outbreak emerged, there had been a nation-wide shortage of hepatitis A vaccines since 2015 [34]. The opportunity of a target vaccination campaign was however discussed at an early stage between the regional health directorate and the ministry of health. The feasibility was also discussed with prevention officers from community-based organizations involved in sexual health promotion and STIs prevention (Aides and Enipse) in MSM. One community-based organization estimated at 500 the number of MSM targeted by their actions in Seine-Maritime department. The regional health directorate set up a first campaign in late March/April 2017 with a stock of 1000 hepatitis A vaccines released by the pharmaceutical industry. As those vaccines expired by April 302,017, this campaign had to be quickly and effectively deployed. In order to avoid as much as possible lost to follow-up, no serological check was performed prior vaccination (in agreement with the ministry of health). One dose was offered during the campaign.

In April 2017, the vaccine was offered: 1) in all STIs clinics during the usual opening hours with additional sessions scheduled in the evening (4 in total), 2) in all vaccination clinics with specific schedule, 3) in a vaccination truck parked at the gay sauna in Rouen during outdoor vaccination sessions (2 sessions), 4) in the premises of one community-based organization (Aides, 3 sessions) in Rouen. In December 2017, supply in vaccine had improved and the vaccine was available in vaccination centers, in STIs clinics and in pharmacies (upon prescription by a general practionner). Two outdoor vaccination sessions were set up in January 2018 at the sauna parking in the vaccination truck. They were initially scheduled early December 2017 but delayed due to logistical constraints (unavailability of medical staff).

The epidemiologists described the characteristics of the vaccinees using a self-administrated standardised questionnaire in order to ensure that those targeted by the campaign had similar sexual practices at risk of hepatitis A as cases. Information collected included 1) socio-demographic characteristics (age), 2) sexual behaviors (place of encounters, sexual practices), 3) reasons for vaccination, 4) channel of information regarding the campaign. The total number of vaccines distributed to the target population was used as proxy to calculate the vaccination coverage rate (based on a target population of about 500 men).

#### Communication campaign toward MSM

**Fig. 1:**
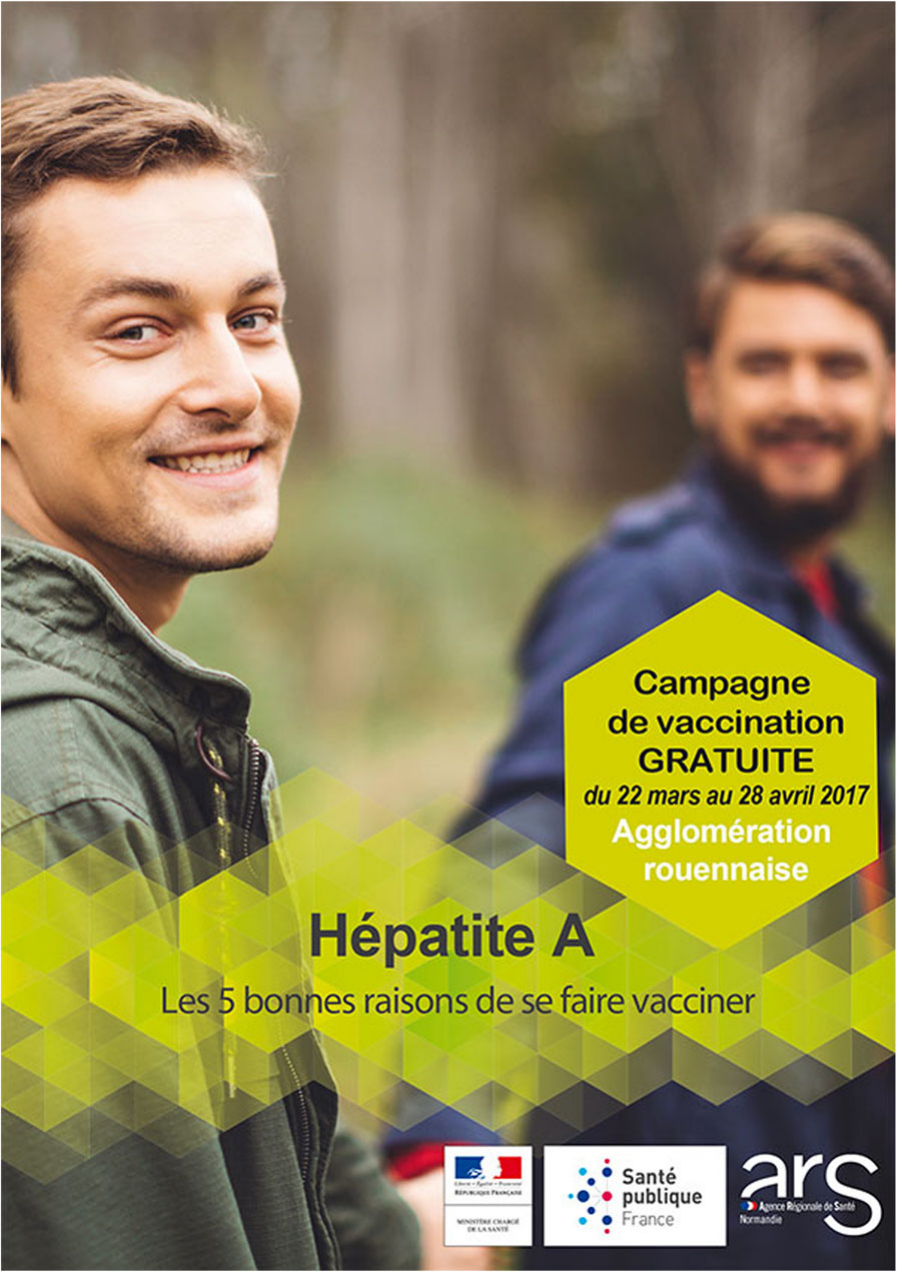
Template of the communication campaign material. Copyright owner: Santé publique France. Reproduction with the permission of Santé publique France

The communication campaign was implemented by the regional health directorate. Experts from the national public health agency designed the communication campaign material (Fig. 1). All the communication material relayed also the schedules of the vaccination sessions.

The campaign targeted the MSM and informed about: 1) the ongoing outbreak in Seine-Maritime department, 2) sexual practices at risk of HAV transmission, 3) effective prevention measures including adherence to good hygiene practices and the availability of an effective vaccine, 4) information about target vaccination sessions for MSM. Poster (*n* = 50) and leaflet (*n* = 500) paper-based campaign were available in sex gay venues (saunas), in all STIs clinics, in the premises of the community-based organizations and during different social events. A digital campaign relaying the same information was launched on the websites and social media accounts of community-based organizations and a news website for MSM (GayViking®).

The communication campaign was deployed by the end of February/March 2017. Communication on social media and on the internet peaked in February/March 2017 and in December 2017. All outdoor vaccination sessions were advertised through pop-up messages on gay-dating apps (Grindr® and Hornet®). Two messages popped up during 24 h the week before the sessions within a distance of 30 miles from Rouen city center. In summer 2017, while the number of cases increased nationally, a national communication campaign was set up in June and August. As part of this campaign, regional pop-up messages promoting hepatitis A vaccination were advertised on Grindr® and Hornet (during summer 2017, stock of vaccines had improved and it was available in all STIs clinics).

The epidemiologists retrospectively collected information on the number of clicks on pop-up messages and the activity of social media account (number of vue, number of posts shared) in order to describe the communication campaign diffusion.

#### Counselling

 Sexual health officers from community-based gay organization delivered counseling on the risk and routes of transmission of hepatitis A infection during sexual intercourses through enhanced face-to-face consultation in gay venues and chating on the net (private discussion on social media). Community-based sexual health organizations reported the observed increase in sexual health counselling sessions during the outbreak period.

### Statistical analysis

Epidemiologists from the regional public health agency entered, cleaned and analysed the data. Descriptive analyses were made using Stata® V14.2 (College Station, TX: StataCorp LP). Proportions and medians were used to describe categorical and continuous variables. Comparisons continuous variables was performed using Wilcoxon test rank.

## Results

### Outbreak description

**Fig. 2:**
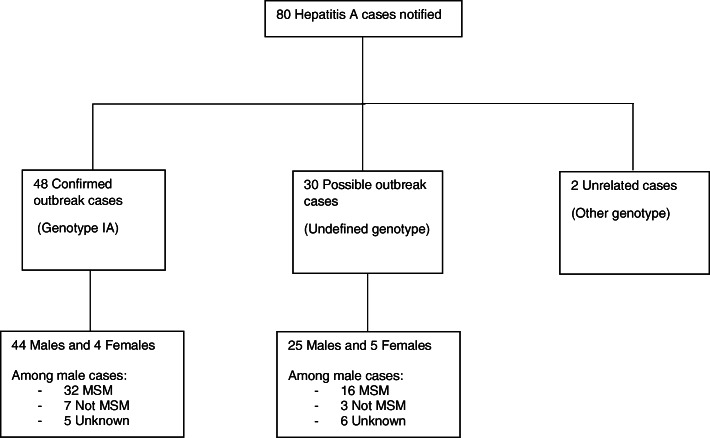
Distribution of Hepatitis A cases according to outbreak status, gender and self-reported sexual orientation, Seine-Maritime department, December 2016 to December 2017. MSM: men who have sex with men

**Fig. 3:**
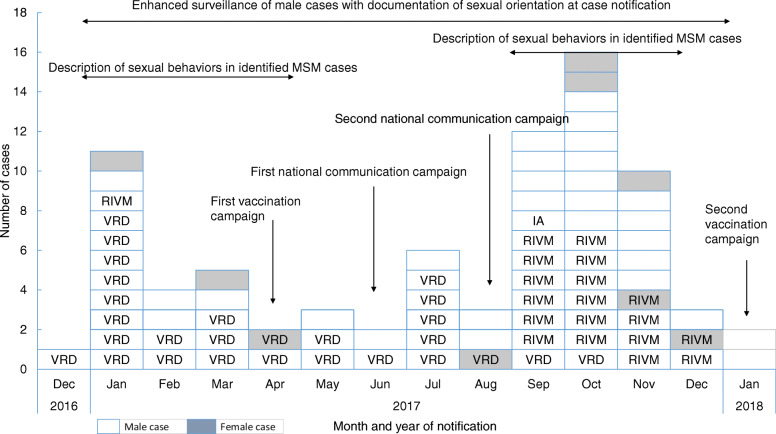
Number of monthly notified confirmed and possible outbreak hepatitis A cases, Seine-Maritime department, December 2016 to December 2017 (*N* = 78). IA: genotype IA, VRD: genotype sequence VRD_521_2016, RIVM: genotype sequence RIVM-HAV16–090

Between December 2016 and December 2017, a total of 80 hepatitis A cases living in Seine-Maritime department, mainly in Rouen area (*N* = 60 (75%)), were notified. There were 48 confirmed outbreak cases (44 men and 4 women), 30 possible outbreak cases (25 men and 5 women) and 2 unrelated outbreak cases (2 men) (Fig. 2). Median age of confirmed and possible cases was 42 years (interquartile interval 25–75% = 27–47, minimum-maximum = 18–70 years). Main characteristics of confirmed and possible cases are reported in Table 1. Among 69 confirmed or possible outbreak cases (88%) in male, 48 declared sexual intercourses with men (79%), 10 were exclusively heterosexual and 11 did not disclose their sexual orientation. Two epidemic waves were observed (Fig. 3). From December 2016 to May 2017, 26 cases were notified and 52 were reported between June and December 2017. Patients were younger in the first wave than in the second one (median ages: 26 versus 42 years, *p* = 0.01). The distribution of the genetic sequence (*N* = 47/48) showed that the first wave cases were almost exclusively infected with the VRD_521_V1 strain (*N* = 19/20) and second waves cases with the RIVM HAV16–090 strain (*N* = 18/27).

**Table 1:** Distribution of main characteristics of confirmed and possible outbreak cases (*N* = 78)

Characteristics	N (%)
Sex	
Male	69 (88%)
Female	9 (12%)
Median age (minimum-maximum)	42 (18–70)
Case status	
Confirmed	48 (61%)
Possible	30 (39%)
Distribution of genetic sequence in confirmed genotype IA cases (*N* = 48)	
VRD_521_V1	28 (60%)
RIVM HAV16–090	19 (40%)
V16–25801	0
Not available	1
Homosexual intercourses during the 2 months previous onset symptoms (MSM) in male cases	
Yes	48 (70%)
No	10 (15%)
Unknown	11 (15%)

From December 2016 to April 2017, 16 out of 20 MSM cases completed the enhanced questionnaire on sexual behaviors (response rate, 80%) during the 2 months preceding the onset of symptoms. Four of them did not report sexual intercourses and their route of contamination remained unknown. Twelve cases reported homosexual intercourses with casual partners (75%) and/or anonymous partners (58%), and/or group sex (50%). Sex partners were encountered mostly through gay-dating apps (67%) and in one sauna (67%) located in Rouen area. Four men reported sexual intercourses abroad (Spain, Luxembourg, Italy, England). From September 2017 to November 2017, characteristics of cases did not differ compared to those characteristics reported from December 2016 to April 2017 (Table 2) (Chi square, *p* > 0.05). Three men reported sex abroad in Israel (*n* = 1) and Spain (*n* = 2).

**Table 2:** Sexual behaviors reported in self-reported MSM cases within 2 months before onset of symptoms, Seine-Maritime department, December 2016 to December 2017

	December 2016 to April 2017	September to November 2017	Total
Response rate	16/20 (80%)	19/28 (67%)	35/48 (72%)
Any sexual intercourse	12 (75%)	19 (100%)	31/35 (89%)
Type of sexual encounter			
Casual partners	9/12 (75%)	12/19 (63%)	21/31 (68%)
Anonymous partners	7/12 (58%)	9/19 (47%)	16/31 (52%)
Sex in group	6/12 (50%)	5/19 (26%)	11/31 (35%)
Mode of sexual encounters			
Gay-dating apps	8/12 (66%)	8/19 (42%)	16/31 (52%)
Sex-on premises (sauna)	8/12 (66%)	12/19 (63%)	20/31 (64%)
Sex abroad	3/12 (25%)	3/19 (16%)	6/31 (19%)
Concomitant sexually transmitted infection			
Self-reported HIV co-infection	7/16 (44%)	4/19 (21%)	11/35 (31%)

A total of 11 men were co-infected with HIV, including 3 HIV diagnosed concomitantly to the HAV infection (Table 2).

### Description of public health control measures

Respectively, 110 and 46 vaccine were administrated during the first and second campaign (Table 3). The vaccination coverage reached 31% in the estimated target population (*N* = 500). During the first campaign, vaccines were administrated mostly in STI clinics (40%) and in the truck parked at the sauna (26%). During the second campaign, vaccines were administrated mostly in in the truck parked at the sauna (67%) and in STIs clinics (33%). Gay dating apps and social media were used to inform about the campaign as they appeared to be very profitable during the first campaign.

**Table 3:** Characteristics of vaccinees, Seine-Maritime department, 2017

	First campaign	Second campaign
Number of vaccine administrated	110	46
Median age (Minimum-Maximum)	36 (18–71)	30 (18–46)
Place of vaccine administration		
STIs clinics	39 (40%)	15 (35%)
Sauna	26 (26%)	30 (65%)
Vaccination centers	19 (18%)	–
Premises of the community-based organizations	12 (12%)	–
Unknown	12	–
Source of information regarding the vaccination campaign		
Gay-dating apps	31 (48%)	33 (72%)
Social media	45 (45%)	13 (38%)
Communication advertised at the sauna	18 (18%)	–
Local gay press	16 (16%)	–
Sauna (Car park)	13 (13%)	–
Gay social event (cinema)	9 (9%)	–
Unknown	12	–

Among the 144/156 vaccinees with available information, 102 (71%) declared that their general practitioner knew about their sexual orientation. However, only 15 (10%) had been proposed hepatitis A vaccination but never get it. The main reason to receive the vaccine was the “fear of the disease” for 89 of them (62%). Eight of them reported being infected with HIV. Vaccinees reported the same sexual practices at risk as cases including several casual partners (75%), anonymous sex (50%) and group sex (32%) and these characteristics were similar to those of cases (Chi square test, *p* > 0.05).

#### Communication campaign

A warning message about the ongoing outbreak was posted by the ENIPSE association on its Facebook® account in March 2017. It was the most shared post related to prevention health in 2017 in Rouen area. The second most shared post was the one advertising the vaccination campaign in April 2017. This association reported a sharp increase in the number of counselling session on prevention of STIs diseases (face to face and chat), from 26 in 2016 to 540 in 2017.

A total of 7 posts were published on Aides Facebook® account on March/April (*n* = 4), December 2017 (*n* = 2) and January 2018 (*n* = 1). Each post was shared 74 to 645 times on March/April 2017 and 235 to 349 times in December 2017/January 2018 which is with the highest range of post sharing.

A local gay journal (GayViking®) also reported Information on the outbreak on its website but no activity data could be retrieved.

Sessions implemented at the car park of the sauna in January 2018 were exclusively advertised through gay-dating apps. Each message broadcasted on the Grindr® social network generated respectively 271 and 99 clicks in April 2017, 41 and 74 clicks in December 2017, 115 and 89 clicks in January 2018. Hornet® did not share activity data.

#### Counselling

One community-based ganization reported a sharp increase in the number of sexual counselling (chat), from 26 in 2016 to 540 in 2017.

## Discussion

We hereby reported an outbreak of sexually transmitted hepatitis A that affected MSM in the Seine-Maritime department (Normandy, France) between December 2016 and December 2017, and the public health measures implemented to control the outbreak. Most cases have been notified in Rouen area. Two epidemic waves were observed with cases mostly affected by the VRD_521_2016 strain during the first wave (until July 2017) and the RIVM HAV16–090 strain during the second wave (August to December 2017). Both strains widely circulated in MSM in Europe in 2017 [18–29]. They were also subsequently reported in other French regions [35]. The outbreak dynamic and the distribution of the genotypes over 2017 suggested that each wave may have had their “own point source”. The initial investigation in January 2017 allowed to link some cases to a common sex party in group. One of the cases who attended that party had sexual intercourses while travelling in Spain [25] and in Paris where the VRD_521_2016 strain was already circulating [36]. The emergence of the RIVM HAV16–090 strain during the second wave remained unexplained. It emerged during the summer and may have been imported from another region in France or another country [29, 35].

The identification of the affected MSM population at an early stage was made easier because one of the first cases spontaneously reported homosexual intercourses with a confirmed HAV partner. It questions the delay and the capability to detect an outbreak sexually transmitted HAV through the current surveillance system. In France, no information on any sexual exposure is routinely collected in the notification form. Sex ratio is routinely used to describe hepatitis A epidemiological data. It allows to suspect sexual transmission in MSM but not to confirm it. Such acute situation requires additional indicators to design and to implement specific prevention measures including counselling and screening of concomitant STIs in cases and their sexual contacts. Therefore, as soon as this outbreak was identified, an enhanced surveillance of hepatitis A cases was implemented. This investigation revealed that almost all outbreak cases had unprotected sex with multiple partners hence a high risk of contracting STIs including HIV [37, 38]. In the specific context of this outbreak, the level of sexual disclosure among cases was high (84%). Therefore, the rationale of excluding sexuality items from mandatory notification in routine could be reassessed [20]. While most of them declared homosexual intercourses, other did not report homosexual intercourses during the exposition period, and other were female. These findings highlighted the risk of secondary dissemination by oro-fecal transmission routes [39] and the mandatory report form should allow the identification of any transmission routes.

Communication and vaccination campaigns were implemented simultaneously. The involvement of the community-based sexual health organization was of invaluable help in that purpose. MSM using gay-dating apps and having sex-on premises (sauna) were specifically targeted as frequently reported by cases [37]. The use of pop-up messages broadcasted on gay-dating apps were of great benefit, as attested by vaccinees that reported being commonly informed through this channel [39]. The high numbers of clicks on each new message certified that information on the campaign was widely diffused, though it is not possible to assess the proportions of vaccinees among viewers of these messages. As also attested by the 7 new cases of HIV infection diagnosed during this outbreak, gay-dating apps should obviously be used more routinely to promote STIs screening [40].

Two vaccinations campaigns were implemented locally at the initiative of the local health authorities. The first campaign in April 2017 did not stop the outbreak. Additional outdoor vaccination sessions at the sauna parking lot were implemented after the upsurge of cases in late summer/autumn 2017. Unfortunately, the second campaign had to be delayed until December 2017/January 2018 due to the unavailability of the truck and shortage in human resources. One third of vaccines were administrated during outdoor vaccination sessions at the sauna. Sex-on premises are location at high risk of hepatitis A transmission [17, 41]. Indeed, although HAV vaccination is free of charge in STIs clinics and vaccination centers, MSM may not spontaneously consult these healthcare centers until they complain about symptoms. In the future, routine vaccination programmes could be proposed there [42]. In addition, the vaccination campaign may have left apart MSM living outside the “gay community”, and very young men, such as students, whose immunity against HAV is probably low [43]. Improved control strategy should involve preventive medicine in university.

In this report, a total of 236 MSM could be considered protected against HAV, 156 by HAV vaccination, and 80 as the result of the disease. Considering an estimated target population of 500 MSM, the final hepatitis A “immunisation” coverage roughly reached 50%. A study by Weerakoona et al. estimated that an immunisation level of 40–50% among MSM was needed to prevent an outbreak especially in young people whose seroprevalence is low [43], whereas some authors pretend the protective level of immunity should rather be 70% [44]. In HIV-infected MSM, the level of immunity should be even higher [45]. Recent seroprevalence study in MSM have been lacking in order to assess the real risk of next outbreak.

After the second campaign less cases were reported. No further geographical cluster were identified. Prospective enhanced epidemiological and virological surveillance in 2018 would have permitted to formally declare the outbreak over in MSM but it was not systematically performed because of resource constraints and because the outbreak was no longer peaking. The situation was also improving at EU level [13]. Public health nurses kept asking about sexual orientation at case notification in order to recommend the vaccine to sexual contact(s) but the information was no longer recorded. Early 2018, access to vaccine was entirely restored in STIs clinics, vaccination centers and in pharmacies (upon prescriptions) and the implementation of vaccine recommendations was much easier than in 2017. Improved access and better adherence of health care professionals to vaccine recommendations are required to increase the vaccine uptake in MSM [46]. While some cases or vaccinees had informed their general practitioner of their sexual orientation, very few had been proposed anti-HAV vaccination. The tension in hepatitis A vaccine supply also refrained the implementation of outbreak control measures [25, 34]. It was a main barrier in the implementation of vaccine recommendations and it reflected the need to develop new strategies in vaccine procurement.

Our study had several strengths. It showed how useful were the different innovative ways in promoting the vaccine in MSM, notably the use of pop-up messages through gay-dating apps in addition to the invaluable role of the community-based organizations [26, 27]. While many authors recommended to improve communication toward MSM and to expand access to vaccine during the outbreak [18–20, 24–28], vaccine offer during outreach campaigns e.g. at sexual venues or in mobile clinics, has been rarely documented [17]. This report described in a very pragmatic how it was done. It also highlighted the challenges in implementing such a campaign with the following 1) the context of vaccine shortage (necessity to release batches), 2) the necessity to reach an agreement on overpassing recommendation from national experts on serological testing before vaccination, 3) the availability of motivated medical staff who participated on a voluntary basis, 4) adhesion of owner of the sauna to the campaign and 5) the implementation of an extensive target communication campaign beforehand. To our knowledge none of the reports previously published had such a step-by-step descriptive approach. On the other hand, there were several limitations to these investigations. The effectiveness of the campaign in controlling the outbreak could not be evaluated. We only ascertained that MSM who received the vaccine had similar characteristics to cases. Characteristics about those MSM who never sought the vaccines remained unknown. Their description would be valuable data in improving acceptance of further vaccination campaigns. The vaccination coverage was a rough estimate (exact size of denominator unknown). A seroprevalence study in MSM remains the only way to assess the risk of further outbreak. Finally we were unable to explain why most of the cases gathered in one main city of the department and not the other.

## Conclusions

The early identification and control of hepatitis A viral outbreaks in MSM is challenging. The control measures described here prevented cases in men with sexual exposures at risk of HAV transmission. These investigation highlighted the importance of getting information on sexual exposure in male hepatitis A cases in order to detect sexually driven epidemic of hepatitis A. Then the implementation of control measures required the involvement of many public health actors. Improving access to the vaccine while offering it for free in location where MSM may be more accessible was essential. The role of community-based organization and modern social networks were even more valuable as they were the main communication channel in the promotion of the vaccination. Cost-effectiveness studies might analyze the interest of a continuous sexual health promotion including vaccination against hepatitis A, hepatitis B and Human Papillomavirus infection in MSM through dating apps and social networks. In routine the prevention of such outbreaks is still threatened by shortages in vaccine supply, and by the low rate of anti-HAV vaccination in the most at-risk populations. This study also highlighted the need to better understand obstacles in HAV vaccine prescription to MSM in general practice, while behavioral surveys might also help to identify levers that can increase vaccine acceptability in MSM population.

## Data Availability

Upon request, extraction of the hepatitis A dataset may be made available from Santé publique France. The request needs the approval of the French data protection authority (CNIL) as restrictions apply to the availability of these data. The other datasets are available upon request.

## Abbreviations

MSM: Men who have sex with men
HAV: Hepatitis A Virus
HIV: Human Immunodeficiency Virus
STI(s): Sexually transmitted infection(s)

## References

[1] Couturier E, Delarocque-Astagneau E, Duponchel JL, Dussaix E, Hoen B, Ichai P (2009). Guide pour l’investigation, la prévention et l’appui à la gestion des cas d’hépatite aiguë A.

[2] Gossner CM, Severi E, Danielsson N, Hutin Y, Coulombier D (2015). Changing hepatitis A epidemiology in the European Union: new challenges and opportunities. Euro Surveill.

[3] Bell A, Ncube F, Hansell A, Davison KL, Young Y, Gilson R (2001). An outbreak of hepatitis a among young men associated with having sex in public venues. Commun Dis Public Health.

[4] Sfetcu O, Ngui SL, Emerson C, McCaughey C, Donaghy P (2011). Hepatitis a outbreak predominantly affecting men who have sex with men in Northern Ireland, October 2008 to July 2009. Euro Surveill.

[5] Stene-Johansen K, Tjon G, Schreier E, Bremer V, Bruisten S, Ngui SL (2007). Molecular epidemiological studies show that hepatitis a virus is endemic among active homosexual men in Europe. J Med Virol.

[6] Urbanus AT, van Houdt R, van de Laar TJW, Coutinho RA (2009). Viral hepatitis among men who have sex with men, epidemiology and public health consequences. Euro Surveill.

[7] Dabrowska MM, Nazzal K, Wiercinska-Drapalo A (2011). Hepatitis a and hepatitis a virus/HIV coinfection in men who have sex with men, Warsaw, Poland, September 2008 to September 2009. Euro Surveill.

[8] Bialek SR, Barry V, Bell BP, Valleroy LA, Behel S, MacKellar DA, Secura G (2011). Seroprevalence and correlates of hepatitis A among HIV-negative American men who have sex with men. Sex Health.

[9] Bouvet E (2005). Sexual practices and transmission of HAV and HCV. Euro Surveill.

[10] Reintjes R, Bosman A, de Zwart O, Stevens M, van der Knaap L, van den Hoek K (1999). Outbreak of hepatitis a in Rotterdam associated with visits to 'darkrooms' in gay bars. Commun Dis Public Health.

[11] Delarocque AE (2001). Epidemic of hepatitis A among homosexual men in Paris, 2000. Euro Surveill.

[12] Vall Mayans M, Benicio SC, Armengol P, Loureiro E (2004). Outbreaks of infectious syphilis and other STIs in men who have sex with men in Barcelona, 2002–3. Euro Surveill.

[13] European Centre for Disease Prevention and Control (2017). Hepatitis a outbreaks in the EU/EEA mostly affecting men who have sex with men – third update, 28 June 2017.

[14] Carrillo-Santisteve P, Tavoschi L, Severi E, Bonfigli S, Edelstein M, Bystrom E (2017). Seroprevalence and susceptibility to hepatitis a in the European Union and European economic area: a systematic review. Lancet Infect Dis.

[15] Coutinho RA, Albrecht-van Lent P, Lelie N, Nagelkerke N, Kuipers H, Rijsdijk T (1983). Prevalence and incidence of hepatitis a among male homosexuals. Br Med J.

[16] Corey L, Holmes KK (1980). Sexual transmission of hepatitis a in homosexual men: incidence and mechanism. N Engl J Med.

[17] WHO position paper on hepatitis A vaccines – June 2012. Wkly Epidemiol Rec. 2012;87((28/29):261–76 https://www.who.int/wer/2012/wer8728_29.pdf?ua=1. Accessed 22 June 2020.

[18] Lanini S, Minosse C, Vairo F, Garbuglia A, Di Bari V, Agresta A (2017). A large ongoing outbreak of hepatitis a predominantly affecting young males in Lazio, Italy; august 2016 - march 2017. PLoS One.

[19] Ndumbi P, Freidl GS, Williams CJ, Mårdh O, Varela C, Avellón A (2018). Hepatitis A outbreak disproportionately affecting men who have sex with men (MSM) in the European Union and European Economic Area, June 2016 to May 2017. Euro Surveill.

[20] Boucher A, Meybeck A, Alidjinou K, Huleux T, Viget N, Baclet V (2018). Clinical and virological features of acute hepatitis a during an ongoing outbreak among men who have sex with men in the north of France. Sex Transm Infect.

[21] Ciccullo A, Gagliardini R, Baldin G, Borghetti A, Moschese D, Emiliozzi A (2018). An outbreak of acute hepatitis a among young adult men: clinical features and HIV coinfection rate from a large teaching hospital in Rome, Italy. HIV Med.

[22] Lin KY, Chen GJ, Lee YL, Huang YC, Cheng A, Sun HY (2017). Hepatitis a virus infection and hepatitis a vaccination in human immunodeficiency virus-positive patients: a review. World J Gastroenterol.

[23] Rodriguez-Tajes S, Perpinan E, Caballol B, Lens S, Marino Z, Costa J (2018). Hepatitis A outbreak in Barcelona among men who have sex with men (MSM), January-June 2017: A hospital perspective. Liver Int.

[24] Raczyńska A, Wickramasuriya NN, Kalinowska-Nowak A, Garlicki A, Bociąga-Jasik M (2019). Acute hepatitis a outbreak among men who have sex with men in Krakow, Poland; February 2017-February 2018. Am J Mens Health.

[25] European Centre for Disease Prevention and Control. Hepatitis A outbreaks in the EU/EEA mostly affecting men who have sex with men, Stockholm, December 2016. https://www.ecdc.europa.eu/sites/default/files/media/en/publications/Publications/13-12-2016-RRA-Hepatitis%20A-United%20Kingdom.pdf. Accessed 22 June 2020.

[26] Werber D, Michaelis K, Hausner M, Wenzel J, Bitzegeio J, Belting A (2017). Ongoing outbreaks of hepatitis A among men who have sex with men (MSM), Berlin, November 2016 to January 2017 – linked to other German cities and European countries. Euro Surveill.

[27] Freidl GS, Sonder G, Bovée L, Friesema I, van Rijckevorsel G, Ruijs Wilhelmina L (2017). Hepatitis A outbreak among men who have sex with men (MSM) predominantly linked with the EuroPride, the Netherlands, July 2016 to February 2017. Euro Surveill.

[28] Beebeejaun K, Degala S, Balogun K, Simms I, Woodhall S, Heinsbroek E (2017). Outbreak of hepatitis A associated with men who have sex with men (MSM), England, July 2016 to January 2017. Euro Surveill.

[29] European Centre for Disease Prevention and Control. Hepatitis A outbreaks in the EU/EEA mostly affecting men who have sex with men, Stockholm, September 2018. https://www.ecdc.europa.eu/en/news-events/epidemiological-update-hepatitis-outbreak-eueea-mostly-affecting-men-who-have-sex-men-2. Accessed 20 June 2020.

[30] Martel M, Erouart S, Vermeulin T. Investigation d’une épidémie d’hépatite A chez des gens du voyage. Institut de veille sanitaire. 2012. https://www.santepubliquefrance.fr/content/download/182155/2306089. Accessed 20 June 2020.

[31] LeBourhis ZM, Roque Afonso E, Vion B, Mathieu A, Ndeikoundam Ngangro N, Nicolay N (2017). Outbreak of hepatitis A among men who have sex with men (MSM), Normandy, France, and December 2–16 to April 2017: implications for targeted vaccination.

[32] Schwarz NG, Revillion M, Roque-Afonso AM (2008). A food-borne outbreak of hepatitis a virus (HAV) infection in a secondary school in upper Normandy, France, in November 2006. Euro Surveill.

[33] Calendrier des vaccinations et recommandations vaccinales 2017 (2017). Ministère des affaires sociales et de la santé.

[34] Haut Conseil de la santé publique. Avis. Actualisation de l’avis du 15 juin 2015 relatif aux tensions d’approvisionnement en vaccins contre l’hépatite A. 19 Mai 2016. https://www.hcsp.fr/explore.cgi/avisrapportsdomaine?clefr=560. Accessed 23 Mar 2020.

[35] Couturier E, Mouna L, Letort MJ, van Cauteren D, Roque Afonso AM, de Valk H (2018). Dix premières années de surveillance de l’hépatite A par la déclaration obligatoire, France, 2006-2015. Bull Epidémiol Hebd.

[36] Penot P, Colombier MA, Maylin S, Molina JM (2018). Hepatitis A infections in men who have sex with men using HIV PrEP in Paris. BMJ Case Rep.

[37] Velter A, Sauvage C, Saboni L, Sommen C, Alexandre A, Lydié N (2017). Estimation de la prévalence du VIH chez les hommes ayant des relations sexuelles avec des hommes fréquentant les lieux de convivialité gay de cinq villes françaises– PREVAGAY 2015. Bull Epidémiol Hebd.

[38] Ndeikoundam Ngangro N, Viriot D, Fournet N, Pioche C, De Barbeyrac B, Goubard A (2019). Bacterial sexually transmitted infections in France: recent trends and patients’ characteristics in 2016. Euro Surveill.

[39] Ruscher C, Werber D, Thoulass J, Zimmermann R, Eckardt M, Winter C (2019). Dating apps and websites as tools to reach anonymous sexual contacts during an outbreak of hepatitis a among men who have sex with men, Berlin, 2017. Euro Surveill.

[40] Friesema I, Sonder G, Petrignani M, Meiberg A, van Rijckevorsel G, Ruijs W, Vennema H (2018). Spillover of a hepatitis A outbreak among men who have sex with men (MSM) to the general population, the Netherlands, 2017. Euro Surveill.

[41] Mazick A, Howitz M, Rex S, Jensen IP, Weis N, Katzenstein TL, Haff J, Mølbak K (2005). Hepatitis A outbreak among MSM linked to casual sex and gay saunas in Copenhagen, Denmark. Euro Surveill.

[42] Tortajada C, de Olalla PG, Pinto RM, Bosch A, Caylà J (2009). Outbreak of hepatitis A among men who have sex with men in Barcelona, Spain, September 2008 – March 2009. Euro Surveill.

[43] Weerakoona A, Chena M, Reada T, Bradshawa C, Fairley C (2012). Immunity to hepatitis a when outbreaks of infection in men who have sex with men (MSM) are rare. Vaccine.

[44] Ali H, Regan DG, Guy RJ, Robertson P, Watchirs-Smith L, McNulty AM (2015). Increasing hepatitis a immunity in men who have sex with men in Sydney, 1996-2012. Vaccine.

[45] Charre C, Ramière C, Roque-Afonso AM, Chidiac C, Zoulim F, Godinot M (2017). Hepatitis A outbreak in HIV-infected MSM and in PrEP-using MSM despite a high level of immunity, Lyon, France, January to June 2017. Euro Surveill.

[46] Petit B, Epaulard O (2020). Men having sex with men and the HPV vaccine in France: a low vaccine coverage that may be due to its infrequent proposal by physicians. Vaccine.

